# 

**Published:** 1902-12-20

**Authors:** 


**IMPORTANT NOTICE.**

**On account of the Christmas Holidays, we beg to announce that we shall go to press with our issue of the 27th DECEMBER on MONDAY NIGHT, the 22nd inst. Advertisements intended for insertion in this issue should therefore reach us by 4 p.m. ON MONDAY, the 22nd inst.**

### NOTICE TO CORRESPONDENTS.

All MSS., Letters, Books for Review, and other matters intended for the Editor should be addressed The Editor, "The Hospital," The Hospital Building, 28 & 29 Southampton Street, Strand, W.C.

The Editor cannot undertake to return rejected MS., even when accompanied by stamped directed envelope.

**Notice to Advertisers and Subscribers.**

All Advertisements, Orders for Copies of The Hospital, and Business Communications should be addressed to **THE MANAGER, (Not to the Editor)**, The Hospital, 28 & 29 Southampton Street, Strand, London, W.C.

The Cost of Subscription, post free, is as follows.—Per week, 2½d.; per quarter, 2s. 3d.; per half-year, 5s. 3d.; per annum, 10s. 6d. Foreign:—Per annum, 15s. 2d., payable, in all cases, in advance.

**NOTICE.—Vol. XXXII. of "The Hospital" (April to September 1902) in handsome cloth binding is now on sale at the office of this Journal, price, 7s. 6d. Cases for binding the Numbers contained in this Volume 1s. 6d. Index to Vol. XXXII., 2½d., post free.**

**The Monthly Part for December is now ready. Price 6d., by post 8d.**

### BOOKS RECEIVED.

BAILLIÈRE, TINDALL AND COX.

"Aids to Gynæcology." By A. S. Gubb, M.D.

"Aids to Forensic Medicine and Toxicology." By W. Murrell, M.D.

PUBLISHED BY AUTHOR.

"Tragedies of Life—a Fragment of To-day." By Rev. T. J. Bass.

### SCRAPS AND GLEANINGS.

An anonymous donation of £100 has been made to the Cancer Research Fund through Sir Alfred Cooper.

The Ladies' Association of the Hampstead Hospital have collected £1,000 in aid of the Building Fund.

A woman in St. Luke's has just died from the effects of pressure on her neck caused by wearing a string of beads.

Medical opinion is to be taken by the Salford Guardians as to the advisability of vaccinating people between 60 and 70 years of age.

At Jarrow there is no apparatus for sending a fire-alarm, and when a fire breaks out the police have to go round and summon each individual fireman.

The members of the Stock Exchange have subscribed over £1,700 towards the fund which Lady Dimsdale has collected on behalf of the Queen Victoria Jubilee Nurses' Institute.

Mr. A. Bailey has given £1,000 in memory of his wife to defray half the cost of the new Victoria Memorial Cottage Hospital at East Grinstead, which has recently been opened by Lady Musgrave.

The Inspector of Reformatories in Ireland reports that dry bread and shell cocoa three times a day, with occasionally a dish of cabbage for dinner, was the sole diet of the children at several industrial schools visited by him last year.

### THE STUDENT OF MEDICINE.

### APPOINTMENTS.

**Harold William Atkinson**, L.R.C.P.Lond., M.R.C.S.Eng., appointed Junior Resident Medical Officer to the North-West London Hospital. **Austin Badcock**, M.B.Lond., appointed House Surgeon to the Brighton Throat and Ear Hospital. **Marcus H. Bulteel**, L.R.C.P.Lond., M.R.C.S.Eng., appointed Surgeon to the Town Hospital, St. Peter Port, Guernsey. **Joseph W. Dawes**, M.B., C.M.Edin., appointed District Medical Officer of the Stoke-upon-Trent Union. **H. Lauder Hamilton**, M.B., Ch.B.Aberd., appointed Resident Medical Officer at the Grafton Street Infectious Diseases Hospital, Liverpool. **Horace Jefferies**, M.R.C.S., L.S.A., appointed Honorary Surgeon to the Bolton Infirmary. **F. W. Lee**, M.R.C.S., L.R.C.P.Lond., appointed Workhouse and District Medical Officer of the Thakeham Union. **Jas. W. Mathewson**, M.B., B.S.Edin., appointed District Medical Officer of the Clun Union. **Percy W. Nicol**, M.D.Edin., D.P.H., R.C.P.S.Lond., appointed District Medical Officer to the Wandsworth and Clapham Union. **Peterswald Pattison**, M.B., Ch.B.Edin., appointed House Physician to the Chester Infirmary. **E. T. Prior**, M.R.C.S.Eng., appointed Medical Officer of the Workhouse of the Loddon and Clavering Union. **W. L. Pritchard**, M.B., C.M., appointed Medical Officer for the Nantyglo District of the Bedwelty Union. **E. Saxton**, L.R.C.P., L.R.C.S.Edin., appointed District Medical Officer of the Stone Union.

**"The Hospital" Institutional Library.**

"Hospitals and Asylums of the World." 4 Vols., with a Portfolio of Plans, complete £12 12s.

"Burdett's Hospitals and Charities, 1902." 5s.

"Cottage Hospitals, General, Fever, and Convalescent." 10s. 6d.

"Hospital Expenditure: The Commissariat." 2s. 6d.

"A Legal Handbook for the Use of Hospital Authorities." 2s. 6d.

"The Cottage Hospital Case Book or Register of Patients." 10s. and 12s. 6d.

"The Furnishing and Appliances of a Cottage Hospital." 6d.

All these are published by the Scientific Press, Ltd., and may be obtained through any bookseller, or direct from the publishers, 28 and 29 Southampton Street, Strand, London, W.C.

